# Do we need more than one Child Perceptions Questionnaire for children and adolescents?

**DOI:** 10.1186/1472-6831-13-26

**Published:** 2013-06-12

**Authors:** Lyndie A Foster Page, Dorothy Boyd, W Murray Thomson

**Affiliations:** 1 Department of Oral Rehabilitation, School of Dentistry, University of Otago, PO Box 647, Dunedin, New Zealand; 2 Department of Oral Sciences, School of Dentistry, University of Otago, Dunedin, New Zealand; 3 Department of Oral Sciences, School of Dentistry, University of Otago, Dunedin, New Zealand

**Keywords:** Children, Indexes, Health status indicators, Validity

## Abstract

**Background:**

In dentistry, measures of oral health-related quality of life (OHRQoL) provide essential information for assessing treatment needs, making clinical decisions and evaluating interventions, services and programmes. The two most common measures used to examine child OHRQoL today are the Child Perceptions Questionnaire at two ages, 8–10 and 11–14 (CPQ_8-10,_ CPQ_11-14_). The reliability and validity of these two versions have been demonstrated together with that (more recently) of the short-form 16-item impact version of the CPQ_11-14_. This study set out to examine the reliability and validity of the Child Oral Health Quality of Life Questionnaires (COHQOL) instruments the CPQ_8-10_ and impact short-form CPQ_11-14_ in 5-to-8-year-old New Zealand children, and to determine whether a single measure for children aged 5–14 is feasible.

**Method:**

A cross-sectional survey was conducted of 5-to-8-year-old children attending for dental treatment in community clinics in 2011. Children were examined for dental caries, with OHRQoL measured using the CPQ_8-10_ and short-form CPQ_11-14_. Construct validity was evaluated by comparing mean scale scores across ordinal categories of caries experience; correlational construct validity was assessed by comparing mean CPQ scores across children’s global ratings of oral health and well-being.

**Results:**

The 183 children (49.7% female) aged 5 to 8 years who took part in the study represent a 98.4% participation rate. The overall mean dmft was 6.0 (SD, 2.0 range 1 to 13). Both questionnaire versions detected differences in the impact of dental caries on quality of life, with the greatest scores in the expected direction. Both versions showed higher scores among those with poorer oral health. There was a very strong and positive correlation between CPQ_11-14_ scores and CPQ_8-10_ scores (Pearsons’s r = 0.98; P < 0.01).

**Conclusion:**

The performance of both versions of the COHQOL measures (CPQ_8-10_ and short-form CPQ_11-14_) appears to be acceptable in this younger age group, and this work represents the first stage in validating this questionnaire in a younger age group. It also further confirms that younger children are capable of providing their own perceptions of oral health impacts. The acceptability of the short-from CPQ_11-14_ in this younger age group lends support to its use in children between ages 5 and 14.

## Background

Dental caries is the most common chronic childhood condition afflicting New Zealanders. It is known to affect children’s development, school performance, and behaviour, as well as affecting their families and the wider community [1,2]. This problem is not unique to New Zealand but is a major health issue affecting children in many countries [3]. Increasingly, investigations of oral diseases and disorders are using clinical disease and psychosocial measures concurrently, recognising the importance of using health-related quality of life evaluations in clinical studies. This has led to a growth in the number and use of condition-specific instruments [4]. In dentistry, measures of oral health-related quality of life (OHRQoL) provide essential information for assessing treatment needs, making clinical decisions and evaluating interventions, services and programmes. Measures currently available for children include the Child Oral Health Quality of Life (COHQOL) questionnaires [5-8], the Child Oral Health Impact Profile (COHIP) [9], the Child-Oral Impacts on Daily Performances (Child-OIDP [10], the Early Childhood Oral Health Impact Scale (ECOHIS) [11], and the Scale of Oral Health Outcomes (SOHO-5) [12].

To date, the COHQOL has been most frequently used; it is a set of scales measuring the negative effects of oral and orofacial disorders on the well-being of 6-14-year-olds and their families. The scales comprise the Child Perceptions Questionnaire at two ages, 8–10 and 11–14 (CPQ_8-10,_ CPQ_11-14_), the Parental-Caregiver Perceptions Questionnaire (P-CPQ) and the Family Impact Scale (FIS). The Child Perceptions Questionnaire at age 6–8 was never developed or validated however, the reliability and validity of the two CPQ versions has been demonstrated [8,13], together with that (more recently) of the short-form 16-item impact version of the CPQ_11-14_[14]. However, the use of two separate measures limits the ability of the CPQ to be used in prospective studies following children through different life stages. By contrast, the COHIP was developed as an instrument for use with children aged from 8 to 18 years. Having a single measure which can be used longitudinally in children over a ten-year age span is a considerable advantage [15].

Measures for children younger than 8 years old remain problematic. Until recently, their OHRQoL was measured using parents as informants, because of concerns that children’s reports would not meet accepted psychometric standards of validity and reliability, largely because of limitations in the respondents’ cognitive capacities and communication skills. Accordingly, the P-CPQ was developed for use with younger children and provides a measure of a child’s OHRQoL. Where both parental and child reports are used, the P-CPQ can be regarded as complementing the latter, thus providing a comprehensive profile of a child’s health and well-being [7]. Until very recently, there has been no self-report measure for children under age 8 because of the methodological and conceptual challenges of developing OHRQoL measures for young children [12]. Around the age of 6 marks the beginning of abstract thinking and self-concept for children [16]. Children start to compare their physical features and personality traits, either with those of other children or against a norm. Their ability to make evaluative judgments of their appearance, the quality of friendships and other people’s thoughts, emotions and behaviours develops through middle childhood [16]. Gradually, by the age of 8, they develop the concepts of time and frequency of event [17]. All of these challenges have led to a lack of measures for children under the age of 8, although the recent development of the Scale of Oral Health Outocmes (SOHO-5) has resulted in a self-report OHRQoL measure for 5-year-old children. The initial assessment showed acceptable reliability and validity in 332 UK children [12].

With the encouraging finding that children as young as five can use a self-report OHRQoL measure, the aim of this study was to determine whether the COHQOL measures (the CPQ_8-10_ and short-form CPQ_11-14_) are reliable and valid in younger New Zealand children, and whether it is feasible to use a single CPQ measure for children aged 5 to 14.

## Method

A survey was conducted of approximately 200 5- to 8-year-old children attending for dental treatment in Hawkes Bay community clinics in 2011. The number of children chosen for this study was to assist in informing sample size determination for a larger multicentre trial. Ethical approval was obtained from the Central Regional Ethics Committee. Consent was obtained from both parent and child before proceeding.

### Sociodemographic characteristics

Information was gathered on each child’s sex, age and ethnicity. The children were categorized into two age groups, with “older” being the 7- and 8-year-olds, and “younger” being the 5- and 6-year-olds. An area-based deprivation measure [18] was used to allocate each participant to a deprivation decile score, based on the residential address. Areas with scores 1 to 3 were classified as “low deprivation”; those with scores 8 to 10 were classified as “high deprivation”.

### Clinical measures

Qualified dental therapists undertook routine clinical examinations, having been trained in the study protocol at the community clinics. Baseline charting recorded for each child included decayed, missing and filled deciduous teeth. Posterior bitewing radiographs were also taken, and these were used to amend the children’s dmft scores appropriately. Intra-examiner reliability was undertaken on twenty films by one experienced clinician who read all the radiographs, with ICC = 0.82.

### OHRQoL measures

Oral health-related quality of life was measured using both the 16-item impact short-form CPQ_11-14_-ISF:16 questionnaire and the 25-item CPQ_8-10_ (Table 1). The overlap in item content between the two questionnaires enabled both versions to be incorporated into a 26-item questionnaire. The reference period used for both was the previous four weeks, as originally used in the CPQ_8-10_ questionnaire [8]. Response options and scores were: ‘Never’ (scoring 0); ‘once or twice’ (1); ‘Sometimes’ (2); ‘Often’ (3); and ‘Every day or almost every day’ (4). An overall CPQ_11-14_ and CPQ_8-10_ score was computed by summing the appropriate item scores for each measure, with a higher score indicating poorer OHRQoL. Test-retest reliability was not examined for either measure. Childrens’ perceptions of their oral health were assessed using two global measures. First, they were asked to rate the health of their teeth and mouth (response options: ‘Very good’, ‘Good’, ‘OK’ or ‘Poor’). Second, they were asked how much their teeth or mouth bother them (response options: ‘Not at all’, ‘A little bit’, ‘Some’ or ‘A lot’). The research assistant administered the questionnaire and read each question to the child.

**Table 1:** **Comparison of item content of the CPQ**_**11-14**_**-ISF:16 and the CPQ**_**8-10**_

**In the past 4 weeks, how often have you (had/been) because of your teeth/mouth**			
**Domain**	**CPQ**_**11-14**_**ISF:16-specific items**	**Items common to ISF:16 and CPQ**_**8-10**_	**CPQ**_**8-10**_**-specific items**
OS^a^		Pain in teeth/mouth	
		Bad breath	
		Mouth sores	Difficulty eating, drinking hot/cold foods
		Food caught between teeth	
FL^b^	Difficulty eating/drinking hot/cold foods	Difficulty chewing firm foods	Trouble sleeping
		Difficulty saying words	Trouble eating foods you like
		Taken longer to eat a meal	
EW^c^		Upset	
		Felt irritated/frustrated	Worried not as good looking
		Felt shy	
		Concerned what people think about teeth/mouth	
SW^d^		Teased/called names	Not wanted to speak/read loud in class
	Argued with children/family	Avoided smiling/laughing	Missed school
		Asked questions	Hard time doing your homework
			Hard time paying attention in school
			Stayed away from activities
			Avoided being with other children
			Avoided talking with other children

^a^Oral symptoms ^b^Functional limitations ^c^Emotional well-being ^d^Social well-being.

Data were analysed with SPSS (version 18.0). The computation of descriptive statistics was followed by bivariate analyses, which used Chi-square tests for comparing proportions; Mann–Whitney or Kruskal-Wallis tests were used (as appropriate) for comparing scores for continuous variables (where these were not normally distributed). Where continuous variables were normally distributed, ANOVA was used to compare means. The alpha value was set at 0.05. Spearman’s rank correlation coefficients were computed to inform the assessment of associations among sociodemographic, clinical and psychosocial characteristics. Pearson’s r was used to examine the correlation between scores on the two scales (CPQ_11-14_ and CPQ_8-10_).

## Results

The 183 5-to-8-year-olds (49.7% female) who took part in the study represent a 98.4% participation rate. Just over half (50.4%) were Māori, and almost half (44.8%) resided in highly deprived areas. The overall mean dmft was 6.0 (SD, 2.0; range 1 to 13). Scores ranged from 0 to 43 for the CPQ_8-10_ and 0 to 37 for the CPQ_11-14_ (Table 2), and these and the domain scores were positively skewed. Both versions detected substantial variability in children’s perception of their OHRQoL, as shown by their scores. Floor effects were substantial for both versions (as evidenced by approximately 14% responding with no impact), while ceiling effects were not apparent. Substantial internal consistency reliability was apparent for each of the two questionnaires (and domains), with that of the CPQ_8-10_ version being superior to that of the CPQ_11-14_ version.

**Table 2:** **Descriptive statistics and internal consistency reliability data for the CPQ**_**11-14**_**, CPQ**_**8-10 **_**and their subscales**

	**Number of items**	**Mean score (SD)**	**Cronbach’s alpha (α)**	**Range of observed scores**	**Percentage with score 0**	**Percentage with maximum score**
CPQ_11-14_	16	6.6 (6.6)	0.83	0 to 37	14.3	0.0
Subscales						
Oral symptoms	4	3.1 (2.9)	0.72	0 to 14	18.2	0.0
Functional limitations	4	2.0 (2.6)	0.65	0 to 12	39.8	0.0
Emotional well-being	4	1.0 (1.8)	0.69	0 to 10	61.1	0.0
Social well-being	4	0.6 (1.5)	0.71	0 to 11	72.1	0.0
CPQ_8-10_	25	7.8 (8.4)	0.87	0 to 43	13.7	0.0
Subscales						
Oral symptoms	5	3.7 (3.5)	0.76	0 to 17	16.9	0.0
Functional limitations	5	2.0 (2.8)	0.71	0 to 14	43.2	0.0
Emotional well-being	5	1.1 (2.0)	0.70	0 to 12	57.9	0.0
Social well-being	10	3.0 (3.2)	0.77	0 to 14	67.0	0.0

**Figure 1:**
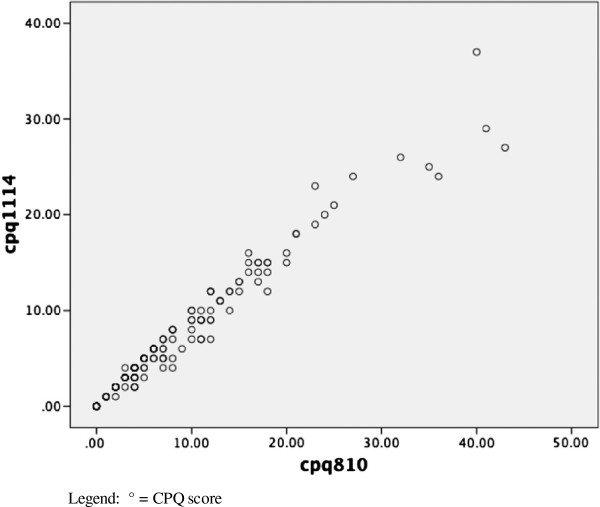
**Scatterplot of scale scores of the CPQ**_**11-14 **_**and CPQ**_**8-10**_**.** Legend: ° = CPQ score.

There was a very strong and positive correlation between scores on the CPQ_11-14_ and the CPQ_8-10_, with a Pearson's r of 0.981 (P < 0.01) for all children, and Pearsons’s r values of 0.983 (P < 0.01) and 0.981 (P < 0.01) for the younger and older categories of children respectively. A scatterplot of scale scores for all children depicts the strength of that association (Figure 1).

Both questionnaire versions detected differences in the impact of dental caries on quality of life, with the greatest scores in the expected direction: children who presented with the highest caries burden had the highest scores (Table 3). The differences for both questionnaires were statistically significant (as were those representing the oral symptoms domain). There was a gender difference in the overall CPQ_11-14_ and CPQ_8-10_ scores, with females scoring higher than males. There was a distinct deprivation gradient with both CPQ versions, with children from families living in highly deprived areas having higher scores than those in less deprived areas.

**Table 3:** **Mean CPQ**_**11-14**_**, CPQ**_**8-10 **_**and domains by sociodemographic characteristics and caries experience (brackets contain standard deviation unless otherwise indicated)**

			**Oral symptoms**		**Functional limitations**		**Emotional well-being**		**Social well-being**	
	**CPQ**_**11-14**_	**CPQ**_**8-10**_	**CPQ**_**11-14**_	**CPQ**_**8-10**_	**CPQ**_**11-14**_	**CPQ**_**8-10**_	**CPQ**_**11-14**_	**CPQ**_**8-10**_	**CPQ**_**11-14**_	**CPQ**_**8-10**_
All children	6.6 (6.6)	8.4 (7.8)	3.1 (2.9)	3.7 (3.5)	2.0 (2.6)	2.0 (2.8)	1.0 (1.8)	2.1 (2.0)	0.6 (1.5)	1.1 (2.4)
Older	7.2 (7.0)^a^	8.4 (8.9)	3.4 (3.0)^a^	4.1 (3.6)^a^	2.1 (2.5)	2.1 (2.8)	1.1 (2.0)	2.2 (2.2)	0.7 (1.7)	1.2 (2.7)
Younger	5.1 (5.5)	6.2 (6.9)	2.2 (2.4)	3.0 (3.2)	1.9 (2.7)	1.8 (2.8)	0.7 (1.1)	1.7 (1.3)	0.3 (0.7)	0.7 (1.4)
Sex										
Male	5.4 (5.1)^a^	6.1 (5.9)^a^	2.8 (2.5)	3.5 (3.2)	1.8 (2.3)	1.5 (2.2)	0.6 (1.0)^a^	1.6 (1.0)	0.4 (1.2)	0.7 (1.8)^a^
Female	7.7 (7.7)	9.4 (10.0)	3.4 (3.2)	4.0 (3.8)	2.2 (2.8)	2.5 (3.2)	1.3 (2.3)	2.5 (2.5)	0.8 (1.7)	1.5 (2.8)
Ethnicity										
NonMāori	6.5 (6.2)	7.5 (7.3)	3.1 (2.9)	3.7 (3.4)	2.1 (2.7)	2.0 (2.6)	1.0 (1.3)	2.1 (1.9)	0.6 (1.3)	1.0 (2.0)
Māori	6.6 (7.0)	8.0 (9.3)	3.1 (3.0)	3.7 (3.6)	1.9 (2.5)	2.0 (2.9)	0.9 (1.9)	2.1 (2.1)	0.6 (1.6)	1.2 (2.7)
NZDep^h^										
High	7.5 (7.9)	9.2 (10.0)	3.5 (3.4)	4.1 (4.1)	2.2 (2.8)	2.3 (3.1)	1.1 (2.1)	2.3 (2.2)	0.9 (2.0)	1.7 (2.9)^a^
Medium	6.0 (5.9)	6.9 (7.3)	2.6 (2.3)	3.3 (2.7)	2.0 (2.5)	1.9 (2.7)	0.9 (1.7)	2.0 (2.0)	0.5 (1.1)	0.7 (2.0)
Low	5.7 (4.2)	6.6 (4.9)	3.2 (2.6)	3.9 (3.3)	1.7 (1.9)	1.6 (1.8)	0.7 (1.0)	1.7 (1.0)	0.1 (0.4)	0.4 (1.1)
dmft tertiles										
Low (dmft ≤ 4)	5.1 (5.3)^ab^	5.9 (6.2)^ad^	2.4 (2.6)^af^	3.0 (3.2)^ag^	1.6 (2.3)	1.5 (2.3)	0.5 (0.9)	1.6 (0.9)	0.6 (1.3)	0.8 (1.6)
Med (dmft 5 + 6)	5.7 (7.3)^bc^	6.8 (9.4)^de^	2.5 (2.7)^f^	3.1 (3.3) ^g^	1.7 (2.3)	1.7 (2.8)	0.9 (2.0)	1.9 (2.1)	0.6 (1.8)	1.1 (3.0)
High (dmft ≥ 7)	7.9 (6.6)^c^	9.5 (8.5)^e^	3.9 (3.0)	4.7 (3.7)	2.5 (2.9)	2.5 (3.0)	1.1 (1.7)	2.2 (1.9)	0.6 (1.4)	1.3 (2.3)

^a^ P < 0.05 Kruskal-Wallis/Mann–Whitney.

^bcdefg^ Superscript letters with the same symbols indicate groups which do not differ significantly by post hoc criteria.

^h^ data missing for 15 children.

Both versions of the CPQ showed higher scores among those with poorer oral health (Table 4), with the differences in CPQ_8-10_ scores being of greater magnitude. Both versions of the questionnaire demonstrated positive, statistically significant, and very similar correlations with self-rated oral health and overall impact on quality of life.

**Table 4:** **Mean CPQ**_**11-14 **_**and CPQ**_**8-10 **_**scores by global oral health questions (SD)**

	**CPQ**_**11-14**_			**CPQ**_**8-10**_		
	**All**	**Older**	**Younger**	**All**	**Older**	**Younger**
Self-rated oral health						
Very good	5.7 (6.5)^a^	6.8 (7.1)	3.3 (4.2)^a^	6.6 (8.2)^a^	7.8 (9.1)	4.1 (5.2)^a^
Good	5.7 (5.5)	6.1 (5.5)	4.7 (5.6)	6.8 (7.0)	7.2 (6.8)	5.6 (7.5)
OK/Poor	8.6 (7.9)	9.0 (8.5)	7.5 (6.0)	10.2 (9.8)	10.7 (10.9)	9.1 (6.8)
Spearman's rho	0.20 ^c^	0.13	0.36 ^c^	0.22 ^c^	0.15	0.36 ^c^
Impact of oral health on quality of life						
Not at all	4.5 (4.9)^b^	5.0 (5.4)^b^	3.3 (3.5)^b^	5.1 (5.9)^b^	5.5 (6.3)^b^	4.1 (4.7)^b^
A little bit	7.8 (7.0)	9.0 (7.4)	3.8 (2.9)	9.2 (8.5)	10.5 (9.1)	4.7 (3.3)
Some/A lot	10.7 (8.1)	10.1 (8.5)	11.9 (7.5)	13.5 (11.3)	13.2 (12.3)	14.2 (9.8)
Spearmans rho	0.34 ^c^	0.34 ^c^	0.45 ^c^	0.40 ^c^	0.37 ^c^	0.46 ^c^

^a^ p-value < 0.05 Kruskal-Wallis.

^b^ p-value < 0.01 Kruskal-Wallis.

^c^ correlation significant at 0.01 level.

## Discussion

This is the first study (to our knowledge) to examine the COHQOL CPQ versions in a younger age group. At the time of this study, no validated self-report OHRQoL measure was available for children of this age. Both versions of the COHQOL measures (CPQ_8-10_ and short-form CPQ_11-14_) appear to be acceptable in this younger age group, and the data further confirm that younger children are capable of providing their own perceptions of oral health impacts. The acceptability of the short-form CPQ_11-14_ in this younger age group lends support to utilising this version from age 5 to 14.

A weakness of this study is that the children comprise a convenience sample of participants in a clinical study, whereby children who required treatment were invited to take part; hence, the findings may not be generalisable. However, a strength is the high participation rate, with 183 of the 186 children invited having consent and assenting to take part. Among the study’s other strengths were that the questionnaire was administered to children by a trained research assistant, and the clinical data collection included radiographic diagnosis of caries giving a more accurate estimate of clinical caries status due to the general underestimation of carious lesions where radiographic diagnosis is not used [19].

The psychometric properties of both versions of the CPQ were found to be acceptable in this younger age group. The single item in the CPQ_11-14_ that is not present in the CPQ_8-10_ version (“Argued with children/family”) is found in the social well-being domain. Despite the former having only 16 items, Cronbach’s alpha was 0.83, well above the arbitrary threshold of 0.7 [20] and only slightly less than the 0.87 observed for the 25-item CPQ_8-10_. Instruments with greater numbers of items tend to have higher alpha values [21], but the 16-item CPQ_11-14_ revealed good internal consistency overall; its social well-being domain had a Cronbach alpha value of 0.71 with only 4 items, while the 10 items in the CPQ_8-10_ version had an alpha of 0.77. Floor effects did appear to be a problem with both versions, with the scores for the emotional and social well-being domains being very high, and the CPQ_11-14_ having the greatest. Whether this is problematic is unclear at this stage; further investigation in other populations and settings is warranted. To further support the performance of these versions, the observed gradients in mean scores for both versions across the categories of the global items show that the concurrent validity was excellent. This means that, even at this young age, children are aware of their poor oral health status and the impact on their quality of life.

Where construct validity is concerned, the associations with oral health were strong and significant and in the hypothesised direction (with poorer oral health in the children with the greatest caries experience). Children with more caries had oral symptoms domain scores which were significantly higher than for those with less caries. This was also the case for the functional limitations domain scores for the CPQ_8-10_ (but not for the CPQ_11-14_). Gradients with caries experience were apparent across scores for all of the domains (except for social well-being in the CPQ_11-14_). A criticism of these findings is that they could be due to the children not understanding the items in either instrument due to the language used. While this is possible, it is unlikely given the consistency of the gradients across the response categories of the global OHRQoL item in Table 4: these suggest strongly that the younger children’s understanding of the items was as good as that of the older children. Thus, while there may be a theoretical objection to using the instrument with younger children, the empirical data do not support it.

There was a difference between the two original versions in the reference period used: the CPQ_8-10_ had been validated with a 4-week reference period and the CPQ_11-14_ validated with a 3-month one. This introduced the challenge of which to use in this younger age group. We opted to use the 4-week reference period because a shorter time frame has been supported for use with younger children, with reference to the previous seven days having been advocated previously [22]. However, as with the development of the SOHO-5, we felt that very few children would have experienced the impact of caries within such a short time frame [12]. The 4-week interval appeared to be acceptable for these younger children.

Developing a valid and reliable OHRQoL measure for young children has important implications because it can enhance understanding of how oral conditions affect the life of younger children. Dental caries is a chronic disease which affects many young children, with 50% of New Zealand 5-year-olds having experienced it [1]. It is thus important to measure how this impacts on children’s day-to-day lives and whether changes in clinical care may affect this. The CPQ versions have shown validity and reliability in older age groups and now appear to show some promise for use in a younger age group.

## Conclusion

It appears that it is practical to use the CPQ_11-14_ with children as young as five years old, although this study represents the first stage in validating this questionnaire for children younger than those for whom it was originally designed. However, further research with population-based samples and in other settings is required in order to confirm the findings from this clinical sample of children with relatively high caries experience. Further work is necessary to determine the appropriateness of the language for the younger age group.

## Competing interests

The authors declare that they have no competing interests.

## Authors’ contributions

All authors have made substantive contribution to this study and manuscript and have reviewed the final paper. All authors read and approved the final manuscript.

## Pre-publication history

The pre-publication history for this paper can be accessed here:

http://www.biomedcentral.com/1472-6831/13/26/prepub
